# The Impact of the Angular Head Movement’s Velocity during Diagnostic Maneuvers on Proper Benign Positional Paroxysmal Vertigo Diagnosis and Therapy

**DOI:** 10.3390/diagnostics13040665

**Published:** 2023-02-10

**Authors:** Igor Anurin, Marlena Ziemska-Gorczyca, Dana Pavlovschi, Ireneusz Kantor, Karolina Dżaman

**Affiliations:** Department of Otolaryngology, Centre of Postgraduate Medical Education, Marymoncka 99/103, 01-813 Warsaw, Poland

**Keywords:** BPPV, repositioning maneuvers, otoliths, angular head movement velocity, Epley maneuver, Dix-Hallpike maneuver

## Abstract

Based on the current state of the BPPV field, there are no guidelines that specify an angular head movement’s velocity (AHMV) during diagnostic maneuvers of BPPV. The aim of this study was to evaluate the impact of AHMV during diagnostic maneuvers on proper BPPV diagnosis and therapy. The analysis covered the results obtained in 91 patients with a positive result of the Dix-Hallpike (D-H) maneuver or the roll test. The patients were divided into four groups based on values of AHMV (high 100–200°/s and low 40–70°/s) and the BPPV type (posterior: PC-BPPV or horizontal: HC-BPPV). The parameters of the obtained nystagmuses were analyzed and compared to AHMV. There was a significant negative correlation between AHMV and latency of nystagmus in all study groups. Furthermore, there was a significant positive correlation between AHMV and both maximum slow phase velocity and average frequency of nystagmus in the PC-BPPV groups, whereas it was not observed in the HC-BPPV patients. Complete relief of symptoms was reported after 2 weeks and was better in patients diagnosed with maneuvers performed with high AHMV. High AHMV during the D-H maneuver allows the nystagmus to be more visible, increasing the sensitivity of diagnostic tests and is crucial for a proper diagnosis and therapy.

## 1. Introduction

Dizziness is a common condition and ranks second (following headaches) in terms of the frequency of reported symptoms [1]. Approximately 8% of patients in General Practitioner’s offices report this symptom. Moreover, 3.3% of all Emergency Department visits are caused by dizziness [2]. In daily neurological practice, patients with dizziness also represent a large group who require a multidisciplinary approach. The complaints reported by them are often a component of various diseases from different medical fields.

One of the most common causes of dizziness is benign positional paroxysmal vertigo (BPPV) [3]. It is estimated that BPPV represents 17–42% of all balance disorders and its lifetime prevalence in the general population is 10% [4]. In 70% of BPPV cases, the cause is unknown but it is confirmed that the risk of BPPV increases with age [5]. Head trauma is the most common cause and is estimated to represent 8.5–20% of all cases [6]. In this group of patients, multicanal and bilateral BPPV are the most common types of BPPV [7,8]. Post-traumatic BPPV can be caused by whiplash injuries, intense exercise, otological surgeries (stapedotomy, cochlear implantation, and others) [9], but also dental procedures [10]. The nature and severity of injuries that can cause BPPV varies considerably from minimal head trauma (being hit on the head with a ball during a game) through moderate to severe head and neck injuries with loss of consciousness [8]. There are no clear and accepted criteria for the diagnosis of post-traumatic BPPV in the scientific literature, nor is there a unified opinion on the time from injury to the onset of symptoms [11].

The latest publications confirm that there is an association between decreased level of vitamin D and occurrence of BPPV. Moreover, patients with recurrent BPPV have significantly lower levels of vitamin D. The recent metanalysis confirmed that vitamin D supplementation reduces recurrences of BPPV [12,13,14].

BPPV is characterized by short-term attacks of vertigo (lasting less than 1 min), sometimes described as dizziness or instability, accompanied by nystagmus and other symptoms, such as periodic nausea or vomiting, drenching sweats, a feeling of fear, or headaches [15,16,17]. The first attack might be more intensive and mimic acute vestibular syndrome or even stroke. Due to the variety of symptoms and their intensity of occurrence, BPPV should be especially differentiated from transient ischemic attack (TIA), acute ischemic stroke, intracranial tumors, and other lesions, especially in the cerebellum and the fourth ventricular region [18,19,20,21]. It is estimated that 20% of BPPV cases may be misdiagnosed [22]. The three-step HINTS algorithm is helpful in the differential diagnosis and includes the Head Impulse Test, an assessment of the Nystagmus and Test of Skew (vertical ocular misalignment) [23,24]. At the beginning of symptoms, HINTS could be even more sensitive in stroke diagnosis than MRI [19,24]. Careful evaluation of nystagmus during diagnostic maneuvers is the basis for differentiating its origin. Such features of triggered nystagmus as change of direction of nystagmus without change of head position, persistent positional nystagmus without a feeling of dizziness, vertical nystagmus (downward or upward) without rotational component, nystagmus inconsistent with the plane of stimulated semicircular canals (e.g., rotational nystagmus during stimulation of the lateral canals), as well as vomiting provoked by only one change of head position without nystagmus or perceived dizziness, strongly suggest a central origin [25].

According to the consensus of the Classification Committee of Vestibular Disorders of the Bárány Society, diagnosis of BPPV is based on diagnostic maneuvers and accurate interview with patient [26]. There are no other diagnostic methods described in the literature, apart from diagnostic maneuvers [15,20,26]. Therefore, the results of diagnostic maneuvers after the initial diagnosis of BPPV based on the patient’s history are a key element in determining further therapeutic management and prognosis. Complete BPPV diagnostic evaluation consists of identifying the involved semicircular canal and determining the mechanism (cupulolithiasis or canalolithiasis) in which these changes arise. By assessing the patient’s medical history, it can be assumed which canal might be affected. Importantly, the nature of nystagmus triggered during the maneuvers is specific to each of the semicircular canals. The D-H maneuver is performed in the case of suspected involvement of the posterior semicircular canal [27]. If the involvement of the lateral semicircular canal is suspected, the supine roll test should be performed [27,28]. The most frequently affected semicircular canal is the posterior semicircular canal; canalolithiasis of the posterior semicircular canal represents approximately 60–90% of all cases [22,26]. The second most common forms of BPPV are canalolithiasis and cupulolithiasis of the lateral semicircular canal (10–20%) [29,30]. Other forms of BPPV, such as posterior semicircular cupulolithiasis, anterior semicircular canalolithiasis, multilateral unilateral and bilateral BPPV, are found rarely.

The treatment of BPPV is relatively simple and effective, based on repositioning maneuvers [26]. They are a first-line treatment; pharmacological treatment is not effective. In PC-BPPV, the most popular and effective is the Epley maneuver; nevertheless, the Semont maneuver may also be performed in the treatment of PC-BPPV. According to a recent randomized clinical trial, both Semont and Epley maneuvers have similar efficacy, but in regard to the severity of dizziness after treatment, the Epley maneuver produced significantly better results than the Semont maneuver did [31,32]. In HC-BPPV cases, both Lempert and Gufoni maneuvers are recommended [20]. Nevertheless, the latest publication shows the new maneuver: the affected-ear-up 90° maneuver, which is similarly effective against lateral canalolithiasis [33]. The affected-ear-up 90° maneuver is easier to perform and more comfortable for patients and thus could be more suitable, especially for elderly people with cervical spine diseases, than other maneuvers. Patients, after successful repositioning, feel nearly immediate relief and vertigo disappears, but the feeling of instability may persist for a few weeks after the repositioning. However, the basis for effective treatment is an accurate diagnosis of BPPV. Although BPPV is one of the leading causes of dizziness, it is still often overlooked in diagnosis, or is misdiagnosed, possibly due to failure to perform or incorrectly perform diagnostic maneuvers.

Based on the current state of knowledge, there are Clinical Practice Guidelines for BPPV diagnosis [20,26], but they do not specify the angular head movement’s velocity (AHMV) during diagnostic maneuvers of BPPV, apart from the general statement “the maneuver must be performed quickly”. In our opinion, this velocity value is crucial for a proper BPPV diagnosis and treatment. Therefore, this study aims to evaluate the impact of AHMV on diagnostic maneuvers’ results and to determine its optimal value.

## 2. Materials and Methods

The prospective study enrolled 243 patients treated for paroxysmal dizziness and balance disorders. The patients were treated at the Department of Otolaryngology, Centre of Postgraduate Medical Education in Warsaw in 2018–2020. Based on the consensus of the Classification Committee of Vestibular Disorders of the Bárány Society [26] and the inclusion criteria described in Table 1, 91 of them have been subjected to further analysis. The remaining 152 people were excluded from the study. In that excluded group were also patients with a multicanal form of BPPV diagnosis due to the possibility of overlapping nystagmus responses and patients diagnosed with BPPV of the lateral semicircular canal cupulolithiasis due to small unrepresentative group.

**Figure 1:**
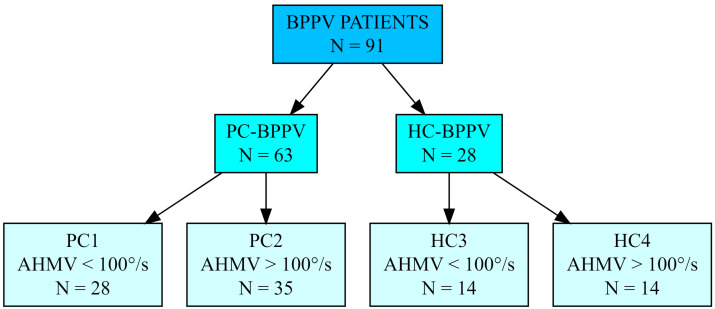
The structure of the study groups.

All 91 patients included in further analyses were divided as follows into 4 groups based on BPPV type and low AHMV (approximately 50°/s; from 40°/s to 70°/s) or high AHMV (over 100°/s; from 100°/s to 200°/s) values of during maneuvers (Figure 1):

Study group I (PC1)—28 patients (30.8%) diagnosed with posterior semicircular canal BPPV (PC-BPPV) and low AHMV. The mean age was 47.9 years. Women represented 68% of the patients (n = 19) and men 32% (n = 9). The average AHMV was 56°/s.

Study group II (PC2)—35 patients (38.5%) diagnosed with PC-BPPV and high AHMV. The mean age was 49.6 years. There were 31 women (89%) and 4 men (11%). The average AHMV was 138.2°/s.

Study group III (HC3)—14 patients (15.4%) diagnosed with lateral semicircular canal BPPV (HC-BPPV) and low AHMV. The mean age was 48.6 years. There were 9 women (64%) and 5 men (36%). The average AHMV was 52.7°/s.

Study group IV (HC4)—14 patients (15.4%) diagnosed with HC-BPPV and high AHMV. The mean age was 46.2 years. There were 11 women (79%) and 3 men (21%). The average AHMV was 145.1°/s.

The D-H maneuver and the supine roll test were performed in all patients. Diagnostic maneuvers were performed manually according to generally accepted assumptions. The presence of triggered nystagmus as a positive test result was recorded using the Frami-VCOR device, consisting of an accelerometer (adapted to measure AHMV) and video googles (allowing the registration of nystagmus) by FRAMIRAL. In the VNG program (version 1.7.10.), the following nystagmus parameters were evaluated: latency, duration, maximum AHMV, average frequency (Average Freq), and the maximum slow phase eye velocity (SPEVmax). The inclusion criteria for the analysis were at least 3 tilts of the eyeball with nystagmus features and duration not shorter than 3 s. The accelerometer measured the speed of the patient’s head movement in two planes depending on which maneuver was performed. The measurements were taken from the beginning of the maneuver to the end of the head’s movement. The results were measured with two decimal places precision. The parameters of the obtained nystagmuses were analyzed and correlated with AHMV. After diagnostic maneuvers, the repositioning maneuvers were performed—Epley or Semont maneuvers were performed in PC-BPPV patients and Lempert or Gufoni maneuvers in HC-BPPV patients. After 2 weeks from therapeutic maneuvers, the therapy effect was assessed using the treatment effectiveness questionnaire (TEQ) and the analysis of persistent nystagmus. The questionnaire consisted of a 5-item subjective treatment efficacy rating scale (0—deterioration, 1—no effect, 2—weak improvement, 3—marked improvement (but dizziness still occurs), 4—complete resolution of complaints).

The obtained results were analyzed statistically: a descriptive statistics, hypothesis testing using the nonparametric test, chi-square test, and the parametric tests, the analysis of variance (ANOVA), Student’s *t*-test, and the Pearson’s r correlation coefficient.

The study was approved by the Bioethics Committee of the Centre of Postgraduate Medical Education in Warsaw (protocol code: 45/PB/2018; date of approval: 11 April 2018).

## 3. Results

**Figure 2:**
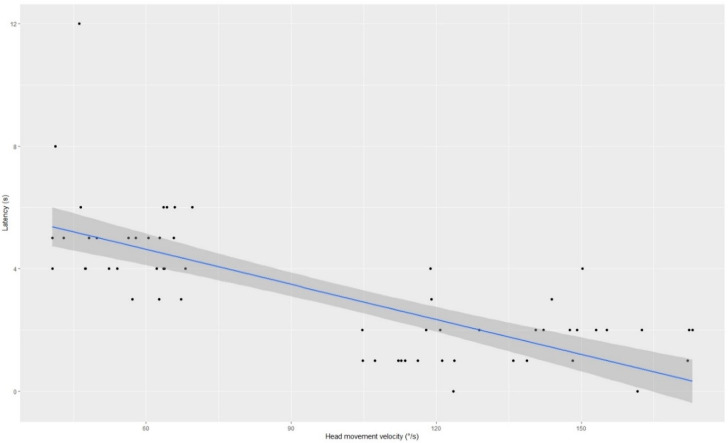
The negative correlation between the latency and the angular head movement velocity during diagnostic maneuvers in all study groups.

**Figure 3:**
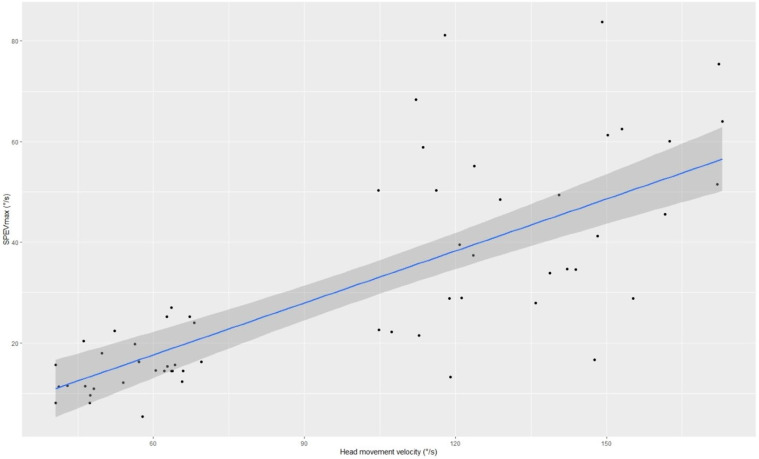
The positive correlation between maximum slow eye phase velocity (SPEVmax) and the angular head movement velocity during diagnostic maneuvers in all study groups.

Taking together all patients, there was a statistically significant negative correlation between AHMV and nystagmus latency in the r-Pearson test (Figure 2). Moreover, there was a statistically significant positive correlation between AHMV and maximum slow phase eye velocity (SPEVmax) (Figure 3), as well as between AHMV and average nystagmus frequency (Average Freq) in the r-Pearson test. The results are shown in Table 2.

If we compared the PC-BPPV groups to each other, the second group (PC2), where the D-H maneuvers were performed faster, had a significantly shorter latency of nystagmus, higher SPEVmax, and higher average frequency of nystagmus compared to the PC-BPPV group with lower AHMV confirmed in the Student’s *t*-test (Table 3). In contrast, we obtained different results comparing patients with HC-BPPV. There was no statistically significant difference between HC3 and HC4 patients in the nystagmus parameters (Table 3).

Additionally, in all study groups, there was no statistically significant correlation between AHMV and nystagmus duration in the r-Pearson test (r = −0.041; *p* = 0.697).

Approximately 2 weeks after the repositioning maneuvers, based on the repeated diagnostic maneuvers and the patient’s subjective assessment, complete symptom relief was found in 94.5% of patients. The effectiveness of the treatment was the highest in the groups with high AHMV during diagnostic maneuvers, respectively, in the PC2 group in 97% vs. 86% in the PC1 (*p* < 0.01) and in the HC4 group 100% vs. 93% in the HC3 group (*p* > 0.05).

**Figure 4:**
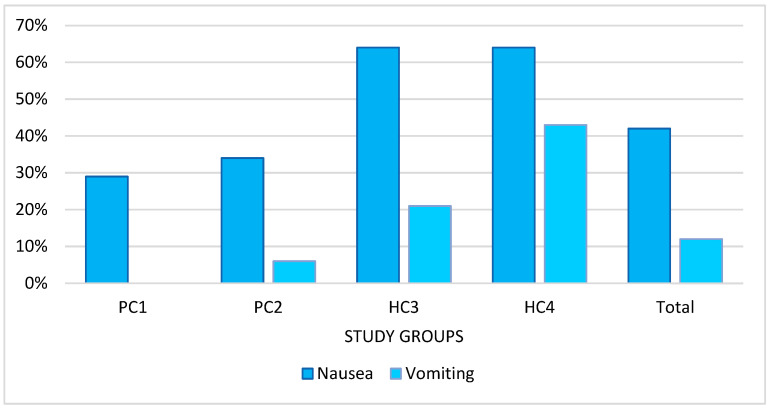
Frequency of nausea and vomiting in the study groups.

In the study, 41.8% of patients reported nausea of varying severity during vertigo attacks and diagnostic maneuvers, while 12.1% of patients also experienced vomiting. Nausea was reported by 28.6% in the PC1 group, 34.3% in the PC2 group, and 64.3% in both the HC3 and HC4 groups. Vomiting was reported by 0% in the PC1 group, 5.71% in the PC2 group, 21.4% in the HC3 group, and 42.9% in the HC4 group (Figure 4).

The analysis shows that in 39% of all cases the duration of the disease was more than a month, and in 56% of cases it was a subsequent episode (recurrence) of vertigo attacks and they did not have diagnostic maneuvers in the past.

## 4. Discussion

Based on a critical appraisal of clinical practice guidelines for the diagnosis and management of benign paroxysmal positional vertigo, there are a couple of elements to improve in guidelines [35]. One of them is that there is a need to specify the velocity of head movement during diagnostic and repositioning maneuvers. Our study provides very important data to improve diagnostic maneuver techniques.

The issue of the relationship between the parameters of stimulation in diagnostic maneuvers in BPPV and the nystagmus reaction has not been clearly defined so far. Most scientific papers discuss the direct effect of gravity on the displacement of otoliths during diagnostic and therapeutic maneuvers [21,26,36,37]. Therefore, the recommendations for the correct performance of diagnostic and therapeutic maneuvers are primarily focused on the angles of tilt and rotation of the head, so that the semicircular canal is located in the plane of gravitational force. Semont was the first researcher who noticed the influence of head movement speed on the displacement of otoliths in the semicircular canal [38]. In 1988, he developed a methodology for the therapeutic maneuver for the posterior semicircular canal, which is known as the Semont maneuver. This maneuver uses both gravity and angular acceleration to rapidly move the head from the affected side to the unaffected side to “throw out” the otoliths of the semicircular canal. In this light, the influence of angular acceleration of head movement on otolith displacement seems to be justified, although it has not been directly proven. In the mathematical model of BPPV, it was confirmed that inertial forces play negligible role in the movement of otoconia during the performance of maneuvers [39].

In the course of this prospective scientific study, the effect of AHMV on the outcome of diagnostic maneuvers for BPPV and its treatment was investigated. An analysis of the effect of AHMV on triggered nystagmus parameters, such as maximum angular velocity of the slow phase of nystagmus, latency, amplitude, frequency, and duration of nystagmus, was performed. In this study, there was a statistically significant negative correlation between AHMV and nystagmus latency when we compared the PC-BPPV patients with AHMV between 40–70°/s during the D-H maneuver (PC1) to the PC-BPPV patients with AHMV between 100–200°/s (PC2). Based on this result, it can be concluded that an increase in AHMV in the case of PC-BPPV reduces the time required for the resistance of displaced otoliths to overcome resistance of endolymph and the elasticity of the cupula [21,26]. Furthermore, other above mentioned nystagmus parameters were lower in the PC1 group compared to the PC2 group. To sum up the above results, in patients with PC-BPPV, it can be concluded that by performing the D-H maneuver at AHMV between 100°/s and 200°/s, we can trigger nystagmus more easily, as the nystagmus is more intense and visible to the examiner. On the other hand, it should be mentioned that dimensions of debris are one of the main factors that influence SPEV. Unfortunately, measurement of otoconia in vivo is unobtainable nowadays. The gravitational forces and viscosity of endolymph also play significant role.

False negative results for the Dix-Hallpike maneuver can be as high as 50% when the D-H maneuver is performed without removal of ocular fixation [40]. Moreover, the sensitivity of diagnostic maneuvers also decreases when patients take drugs suppressing the vertiginous symptoms of vestibular vertigo (e.g., benzodiazepines and antihistamines) [41].

On the other hand, there was no such a correlation in the groups of HC-BPPV patients (HC3, HC4). According to the recommendations of the Barany Society, both the D-H maneuver and the supine roll test should be performed quickly. The results presented in this study indicate that there is no effect of AHMV in the supine roll test on the parameters of the obtained nystagmus. Such a result may be due to several issues in the study. According to the concept of Baloh et al., resulting from their study in 13 patients, in cases of lateral semicircular canalolithiasis, when the head is rapidly rotated toward the occupied canal, the otoliths are accelerated toward the cupula under the influence of angular acceleration of head movement and gravitational forces [42]. The authors reported that rapid head movement in the supine roll test lasting from 200 msec to 300 msec causes an immediate increased positional nystagmus. In our opinion, these discrepancies may be related to different stimulation parameters. In the Baloh et al. study, the AHMV was about 300–450°/s [42]. It is very difficult to achieve such velocities during maneuvers because of the significantly higher incidence of vomiting patients in HC-BPPV than in PC-BPPV [22]. In such situations, some patients may refrain from further diagnostic and therapeutic maneuvers. Epley recommended premedication with scopolamine or diazepam one hour before repositioning treatment [43]. However, current recommendations do not recommend routine premedication, except in cases where severe nausea and/or vomiting occurred during diagnostic maneuvers [20,44]. In our study, patients were instructed on the procedure prior to the diagnostic maneuvers and were warned about the possibility of temporary aggravation of symptoms and the occurrence of nausea or vomiting during their performance.

Another cause of differences between presented results and results of the study by Baloh et al. could be the position of otoliths relative to the cupula in the lateral semicircular canal. The mechanism of canalolithiasis also plays an important role, which could be associated with the slowly moving otoliths, the increasing otolith viscosity, or plugging the semicircular canal [45,46]. An additional important fact is the anatomy of the horizontal canal, which is the shortest semicircular canal. It measures from 12 to 15 mm; in comparison, the posterior semicircular canal measures from 16 to 22 mm [47,48]. It is a significant difference, and as a consequence, it is more difficult to achieve the effective velocity of the otoliths needed for their proper movement. The last but not least issue is the small group of patients presented by the authors [42]—only 13 in comparison to 28 HC-BPPV patients in our study which could impact the final analysis. Thus, further studies on a larger study group are needed in this regard.

To our knowledge, this is the first study that has investigated the impact specific values of AHMV on nystagmus parameters in diagnostic maneuvers in BPPV patients. Nevertheless, in the study by Hwang et al., the effectiveness of the Gufoni maneuver was assessed depending on the speed of its performing in patients with apogeotropic HC-BPPV [49]. However, the measurement of angular speed was not applied in this study.

Treatment of BPPV is extremely simple and effective. In the literature, the success rate of treatment maneuvers is reported to be 84–92% [5,23,50,51]. In this clinical study, follow-up diagnostic maneuvers were performed, and, in addition, patients made a subjective assessment of the treatment effectiveness approximately 2 weeks after repositioning maneuvers. At that time, complete relief of symptoms was found in 94.5% of all patients. The slightly higher success rate than reported by other authors is indicative of the study’s well-chosen inclusion and exclusion criteria and the performance of diagnostic and therapeutic maneuvers by strictly following the guidelines. Moreover, the statistically significant difference in the effectiveness of treatment between PC1 and PC2 may be due to more efficient movement of otoliths in the semicircular canals during the D-H maneuver, which is also the first stage of the Epley maneuver. There is one more important observation, which is that the more intense the nystagmus obtained during the D-H maneuver, the more we can expect greater effectiveness of the Epley maneuver performed in the next stage. It should be mentioned that BPPV patients may spontaneously recover without treatment. It takes average an average of 7 days in HC-BPPV and 17 days in PC-BPPV [23]. Moreover, HC-BPPV has a higher rate of spontaneous resolution than PC-BPPV [52]. However, patients who have had repositioning maneuvers recover faster; the results of the meta-analysis recommend maneuvers rather than conservative treatment [53]. According to the von Brevern study, only 1/3 of patients suffering from BPPV undergo diagnostic maneuvers [54]. These results show that the vast majority of patients do not receive proper treatment. Therefore, it is important to perform diagnostic maneuvers on patients with the suspicion of BBPV [52].

Although better results are obtained with higher AHMV, rapid diagnostic maneuvers can be dangerous for elderly patients and patients with vertebrobasilar instability, obesity, pathological changes, and limitation of spinal mobility, which are also associated with age. In such cases, it is necessary to make a reduction of AHMV, which is associated with lower sensitivity of diagnostic maneuvers. It is safer for a patient and associated with a lower risk of side effects such as cervical spine problems and exacerbation of other chronic diseases. Moreover, in HC-BPPV patients, higher AHMV can provoke canalith jam. On the other hand, in some patients, it would be useful to use semi-automatic mechanical alternatives, such as the Epley Omniax rotator or the TRV chair, which, by immobilizing the head, eliminate hyperextension of the cervical spine [55]. Multi-axial repositioning chairs may also be beneficial for patients with refractory BPPV [56].

## 5. Conclusions

As we noticed, the increased AHMV during performance of the diagnostic maneuver has clinical implications. This allows the nystagmus to be more visible to the examiner, significantly increasing the sensitivity of diagnostic tests. Moreover, performing the D-H maneuver with AHMV between 100 and 200°/s might result in increased effectiveness of the Epley maneuver. To conclude, this study shows that a quick performance of the D-H maneuver is crucial for a proper BPPV diagnosis and therapy.

Performing repositioning maneuvers contributes to the improvement of the functional status of patients, regardless of the form of BPPV and the duration of the disease, which was confirmed on the basis of the assessment obtained after the analysis of the TEQ.

## Figures and Tables

**Table 1:** Inclusion and exclusion criteria.

Inclusion Criteria [26]	Exclusion Criteria
Recurrent attacks of positional vertigo or dizziness caused by lying down or turning in a supine position, lasting up to 1 min	Aged over 60
A positive result of the D-H diagnostic maneuver and/or the supine roll test	Taking CNS inhibiting drugs
A conversion of the D-H maneuver or supine roll test from positive to negative after performing repositioning maneuvers	Neurological diseases
Nystagmus and dizziness cannot be attributed to other abnormalities	Active middle ear inflammation (otitis media) or sudden hearing impairment
The correct result of the HINTS algorithm	Ophthalmic diseases or eyes conditions that influence the VNG results
	HST positive (latent spontaneous nystagmus of peripheral origin [34])
	In the VNG test—uncompensated reduction in caloric excitability of the peripheral vestibular system (>25%)
	In the D-H maneuver—positional nystagmus lasting more than 40 s
	In static–dynamic tests—abnormal result, suggesting a recent peripheral vestibular deficit.

HINTS algorithm (the Head Impulse Test, an assessment of the Nystagmus and Test of Skew), HST—(Head Shaking Test or Head Shaking Nystagmus Test), the D-H maneuver (Dix-Hallpike maneuver), CNS—central nervous system.

**Table 2:** The correlation between the angular head movement velocity and the nystagmus parameters in all study groups.

Parameters	Latency (s)	SPEVmax (°/s)	Average Frequency (Hz)
Average	2.53	7.26	2.94
r-Pearson	−0.47	0.51	0.40
*p*-value	0.00	0.00	0.00

SPEVmax—maximum slow eye phase velocity.

**Table 3:** The correlation between the angular head movement velocity (AHMV) and the nystagmus parameters in PC-BPPV (PC1, PC2) and HC-BPPV (HC3, HC4) groups.

	PC1 (n = 28)	PC2 (n = 35)	*p*-Value	HC3 (n = 14)	HC4 (n = 14)	*p*-Value
Average AHMV (°/s)	56.00	138.24	0.00	52.74	145.08	0.00
SPEVmax (°/s)	15.49	45.16	0.00	38.84	39.99	0.89
Average frequency (Hz)	2.29	3.37	0.00	3.07	3.02	0.83
Nystagmus duration (s)	10.96	10.60	0.72	11.64	11.21	0.72
Latency (s)	5.00	1.49	0.00	1.07	1.64	0.16
Amplitude SPEVmax (°)	5.25	6.54	0.03	9.67	10.67	0.58
Effectiveness of treatment (%)	85.7	97.1	0.00	92.9	100	0.29

## Data Availability

Not applicable.
